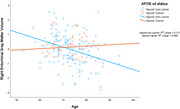# Interaction Between APOE ε4 Status and Age on Medial Temporal Lobe Structures in Hispanic/Latino Older Adults

**DOI:** 10.1002/alz70857_107523

**Published:** 2025-12-25

**Authors:** Rachel Membreno, Carlos E.E. Araujo Menendez, Ariana M Stickel

**Affiliations:** ^1^ San Diego State University, San Diego, CA, USA; ^2^ SDSU/UC San Diego Joint Doctoral Program in Clinical Psychology, San Diego, CA, USA

## Abstract

**Background:**

Apolipoprotein E4 (APOE ε4) is linked to increased genetic risk for Alzheimer's Disease (AD). However, more recent research has found that APOE ε4 may present differently across various racial and ethnic populations, and it is unclear how APOE ε4 status impacts brain aging in underserved groups, such as Hispanic/Latino adults. Thus, we aim to examine the interaction between APOE ε4 and age on the hippocampus and entorhinal cortex in Hispanic/Latino adults.

**Methods:**

We analyzed data from 187 Hispanic/Latino adults (53–94 years; mean = 73 years SD = 7.3) using the National Alzheimer's Coordinating Center database. Our primary exposure variables were APOE ε4 status (ε4 carriers vs. non‐carriers) and age (continuous variable). Brain measures of interest included hippocampal and left and right entorhinal cortex gray matter volumes. Hippocampal and entorhinal cortex volumes were residualized for the intracranial volumes. We examined the interaction between APOE ε4 status and age on brain volumes in medial temporal regions using general linear models adjusted for sex.

**Results:**

There was a significant interaction between APOE ε4 status and age on right entorhinal cortex gray matter volumes (F(1, 170) = 6.576, *p* = 0.011). Specifically, older age was associated with lower right entorhinal gray matter volumes for APOE ε4 non carriers whereas, we did not observe association between age and volumes among APOE ε4 carriers. We did not observe any other significant interactions.

**Conclusion:**

Our cross‐sectional findings suggest that older adults who are APOE ε4 carriers may have better preservation of gray matter volumes in the right entorhinal cortex compared to APOE e4 non‐carriers. Additionally, researchers have suggested that genetic ancestry may influence the relationships between APOE ε4 status and cognitive aging among Hispanic/Latino adults. Future investigations could explore genetic ancestry, other genes, and the APOE ε4 allele to develop a deeper understanding of genetic risk and protection for brain structure and AD in Hispanic/Latino adults.